# Urban-Rural Inequality of Opportunity in Health Care: Evidence from China

**DOI:** 10.3390/ijerph18157792

**Published:** 2021-07-22

**Authors:** Chao Ma, Ze Song, Qingqing Zong

**Affiliations:** 1 School of Economics and Management, Southeast University, Nanjing 211189, China; machao@seu.edu.cn; 2 School of Economics, Nankai University, Tianjin 300071, China; benz1985@nankai.edu.cn; 3 School of Public Economics and Administration, Shanghai University of Finance and Economics, Shanghai 200433, China

**Keywords:** equality of opportunity, health care, fairness gap, urban-rural integrated medical insurance system

## Abstract

(1) Background: We aim to measure the urban-rural inequality of opportunity in healthcare in China based on the theory of Equality of Opportunity (EOp). (2) Methods: Following the *compensation principle*, we establish a decomposition strategy for the *fairness gap*, which we use for the measurement of the inequality of opportunity in urban-rural healthcare utilization. We then use China Health and Nutrition Survey (CHNS) data from 1997 to 2006 to calculate the *fairness gap*. (3) Results: Empirical analysis using CHNS data shows that the ratio of the *fairness gap* to the directly observed average urban-rural difference in healthcare was 1.167 for 1997–2000 and 1.744 for 2004–2006. The average urban-rural difference observed directly from original statistical data may have underestimated the degree of this essential inequity. (4) Conclusions: Our findings suggest that upgrading urban-rural reimbursement ratios may not be sufficient in eliminating the inequality of opportunity in healthcare utilization between urban and rural residents. Within the context of an urban-rural dualistic social structure and widening of the urban-rural income gap, a shift to a pro-disadvantaged policy will be a more effective approach in promoting equality of opportunity in healthcare.

## 1. Introduction

Chinese medical insurance systems are urban-rural dualistic—the Urban Residents Basic Medical Insurance (URBMI) is only for urban residents, and the New Cooperative Medical System (NCMS) is only for rural residents. Owing to deficiency in urban-rural dualistic medical insurance systems, there are large health and healthcare inequalities between urban and rural in China. In order to reduce inequalities in the healthcare system, China recently initiated the Urban-Rural Integrated Medical Insurance System (URIMIS). The URIMIS is still in the exploration stage. It aims to realize equality by unifying the two medical insurance systems.

This divide is evident in two areas: outcome equality and Equality of Opportunity (EOp). Outcome equality means the same reimbursement policy or the same healthcare utilization between urban and rural residents. Most healthcare research relies on outcome equality [1,2,3]. However, due to a large gap in individual and circumstance characteristics between urban and rural residents, it may lead to an inefficiency. (We have provided several examples in the Appendix A as a simple explanation.) EOp means that primary goods (according to Rawls (1971), primary goods mainly include rights, liberties and opportunities, income and wealth, and the social bases of self-respect) should be equally accessible to all individuals, regardless of their race, religion, or other factors that represent their identity [4]. Roemer [5,6,7] refined EOp for empirical studies. EOp is of vital importance for both academic research and policy making [8].

Referring to equality of opportunity, Daniels [9,10] analyzed health inequality. Zheng [11] introduced the income-health matrix to measure health opportunity and inequality in health security *circumstances* and socioeconomic structure. Using data from the UK National Child Development Study, Rosa Dias [12] found significant inequality of opportunity in health. *Circumstances* can affect the self-assessed health level in adulthood directly and indirectly (e.g., through effort such as education), such as parental socioeconomic status (SES) and childhood health. Rosa Dias [13] further improved the measurement of inequality of opportunity by combining Roemer’s framework with the Grossman model of human capital and health demand, and discussed the partial-*circumstance* problem. Based on *circumstances* of childhood condition, Jusot et al. [14] and Trannoy et al. [15] researched the inequality of opportunity in adult health and found that childhood *circumstances*, such as parents’ SES, can largely explain the equity of the health status. Balia and Jones [16] investigated the inequality of opportunity in mortality risk among individuals who (and whose parents) smoke or have smoked. Jones et al. [17] primarily analyzed the role of education in the inequality of opportunity in health, and noted that there are significant and economically sizable linkages between the quality of education and health in some dimensions.

In spite of existing studies, none have focused on China. Based on the theory of the EOp [5,6,7] and the *compensation principle* for the EOp [18,19], this paper calculates and decomposes the urban-rural health care *fairness gaps* in China. Using data from the China Health and Nutrition Survey (CHNS), the results show that: (1) during the two periods of 1997–2000 and 2004–2006, urban-rural differences in healthcare utilization were 225.096 and 268.149, respectively. The urban residents are superior in both periods. Urban-rural differences in SES variables, income, and education are huge too, as well as policy variable actual reimbursement ratio. Moreover, the *fairness gaps* (when we take urban *circumstances* as the “baseline” reference *circumstances*) are 1.167 and 1.744, respectively. These indicate that the results are underestimated from the original statistical data; (2) upgrading the urban-rural reimbursement ratios is probably not sufficient to eliminate the inequality of opportunity in healthcare utilization between urban and rural residents. Under the urban-rural dualistic social structure background, a pro-disadvantage policy will be more effective to promote the equality of opportunity in healthcare.

The rest of this paper is organized as follows: Section 2 presents theories and methods. Section 3 outlines data sources and variables. Section 4 calculates and explains the urban-rural fairness gaps in healthcare by using the CHNS data. Section 5 offers a conclusion.

## 2. Theories and Methods

### 2.1. Equality of Opportunity

In the second principle (the first principle is about the priority of freedom, namely, it should be first considered, on the premise that all people have equal freedom, to maximize the freedom that each one can enjoy) of justice, Rawls [4] points out that public opportunities should be open to all individuals equally, regardless of race, religion or other identity. The difference principle (or Rawls’ maximin principle) means the most disadvantaged group should be granted maximal opportunity. Based on Rawls’ idea [4], Sen [20,21] emphasized that people have the capabilities to choose the lifestyle that has the most value. Dworkin [22,23] introduced the concept of equality of resources, suggesting that some disadvantages should be compensated for, even if they are caused by external factors. Arneson [24] and Cohen [25] modified Dworkin’s theory, and put forth two conceptions: equality of opportunity for welfare and equality of access to advantage. Based on the above, Roemer [5,6,7] proposed an axiomatic approach for EOp empirical studies.

According to EOp, one’s *advantage* (*y*) is determined by two categories, i.e., *circumstances* (*c*) and *effort* (*e*); the former is out of one’s control, the latter is not. *Circumstances* are classified into J *types*. The function is as follows:

(1) $$y_{i}=y(c_{i},e_{i}).$$

The social objective could then be to maximize the *advantage* of the individual that is worst off, i.e., that has the lowest level of *advantage* in a fair society [6]. It is worth noting that Roemer puts forward a somewhat different proposal from that of Rawls, who tries to maximize the minimum level of *advantage* across all individuals, regardless of their type. This maximin criterion is the natural extension of egalitarianism if one is also concerned about efficiency. Indeed, it implies that inequalities are only acceptable if they are to the *advantage* of the worst-off in society:

(2) $$\max\;\underset{c}{\min}\;y(c,\tilde{e}).$$

where $\tilde{e}$ is one’s effort.

Summing the *advantage* of all individuals at each level of *effort*, we obtain:

(3) $$\max\int_{e}\underset{c}{\min}y(c,e)f(e)de,$$

where *f (e)* is the density function of the *effort*.

Roemer [6] repeatedly emphasized that part of the effort can be affected by *circumstances*, which will indirectly affect the distribution characteristics of the *advantage*. It means that the *advantage* is with the (absolute) level of *effort* rather than the (relative) degree of effort in one’s own *type*. However, individuals only take responsibility for degree of effort in their type, not for the distribution characteristics of the effort. Thus, Roemer [6] defines one’s degree of effort using the quantile π in the conditional distribution of their type. Function (3) can be rewritten as (4), which can also be regarded as an explanation of the *maximin principle* of Roemer:

(4) $$\max\int_{\pi }\underset{c}{\min}y(c,\pi )d\pi .$$

### 2.2. Empirical Strategy

#### 2.2.1. Reward Principle and Compensation Principle

For the EOp analysis, Fleurbaey and Schokkaert [18,19] proposed two principles—the *reward principle* and the *compensation principle* under selective egalitarianism.

The *reward principle* encourages inequalities caused by *effort*. For this reason, when measuring the EOp, influences from *effort* should be wiped off firstly. The typical method is to calculate the “corrected” *advantage ỹ_i_* of individual *i* by fixing the value of *effort ẽ*, i.e., *ỹ_i_* = *y*(*c_i_*, *ẽ*). We can obtain the *direct unfairness* by calculating the inequality in *ỹ* using traditional methods such as Gini index.

*The compensation principle* suggests that the inequalities caused by *circumstances* should be compensated. Whatever the *circumstance*, individual should attain the same *advantage* with the same *effort*. Meanwhile, compensation should be given to those who attain less *advantage*. This principle has a close relationship with horizontal equity, which indicates that the same health need should be distributed with equal healthcare, regardless of income level, region, race, etc. All of these factors belong to *circumstances*. Based on the *compensation principle*, it sets a “baseline” *c_i_* (c *), and then we obtain a fair distribution of *yi* (*y_i_* *) via *yi* * = *y*(*c* *, *e_i_*). The unfair inequality of opportunity (the *fairness gap*) is (*y_i_*-*y_i_* *).

Though the two principles and their corresponding methods partially overlap, they are only compatible under certain situations where c and e are completely independent, i.e., they are additively separable [18]. Therefore, empirical assessment needs to choose appropriate principles.

This paper will follow the *compensation principle*. First, we care about how to reimburse rural residents for their *disadvantage* of *circumstances*. This is much closer to the *compensation principle*. Second, while the *reward principle* is usually used to explain inequalities within a certain group, the *compensation principle* is between two groups. We are concerned with the equality in healthcare needs and accessibility between urban and rural residents, which favor the *compensation principle*.

#### 2.2.2. When Roemer Meets Oaxaca

We define c as the indicator of household register (*hukou*). If c is equal to 1, it means individual is an urban resident, while 0 indicates a rural resident. During the analysis, we define all other factors for vector *e*, which is classified into two components, $e^{1}$ and $e^{2}$. Vector $e^{1}$ is on behalf of factors whose correlation with *c* will bring about illegitimate urban-rural differences, e.g., income level and medical insurance types, etc. Vector $e^{2}$ is on behalf of factors that will not bring about illegitimate differences, e.g., health care needs. The *advantage* can be expressed as a function of *c*, $e^{1}$ and $e^{2}$:

(5) $$hc_{i}=\alpha +\beta \varphi (c_{i})+\gamma \psi (e_{i}^{1})+\delta \chi (e_{i}^{2})+\varepsilon_{i},$$

where *hc* is the health care use, *β*, *γ* and *δ* are parameters, α is the constant, and *ε_i_* is an error item. In accordance with the definition of $e^{1}$, it is appropriate to regard $e^{1}$ as a function of c and *π* (the degree of effort), i.e.,

(6) $$e_{i}^{1}=\eta (c_{i},\pi_{i}^{1}).$$

Thus, (5) can be rewritten as:

(7) $$hc_{i}=\alpha +\beta \varphi (c_{i})+\gamma \psi \circ \eta (c_{i},\pi_{i}^{1})+\delta \chi (e_{i}^{2})+\varepsilon_{i}.$$

A more general form can be written as:

(8) $$hc_{i}=\alpha +\beta \varphi (c_{i})+(\gamma +\mu c_{i})\psi \circ \eta (c_{i},\pi_{i}^{1})+(\delta +\rho c_{i})\chi (e_{i}^{2})+\varepsilon_{i},$$

where we add *μ* and *ρ* to separately express the coefficient differences of $\psi \circ \eta \left(c_{i},\pi_{i}^{1}\right)$ and *χ*($e_{i}^{2}$) between urban and rural groups.

Defining *φ*(1) = 1, *φ*(0) = 0, and taking urban *circumstances* (U) as the “baseline” reference background (*c* = 1), we obtain the fairness gap between urban and rural residents. According to Fleurbaey and Schokkaert (2009), the *fairness gap* should be *y*(*c_i_*,*e_i_*)-*y*(*c* *,*e_i_*). However, in order to obtain positive values of the *fairness gap* and its components, we use the reverse value here. Since *y*(*c* *,*e_i_*) and *y*(*c_i_,e_i_*) are the same for urban residents due to the construction of equation, this *fairness gap* in effect is the difference between the counterfactual estimate of the rural residents’ healthcare expenditure in the urban *circumstances* and the actual healthcare expenditure of the rural residents:

(9) $$f.g.=\hat{\beta }+\hat{\rho }\chi (e_{i}^{2}|R)+\hat{\gamma }[\psi \circ \eta (U,\pi_{i}^{1}|R)-\psi \circ \eta (R,\pi_{i}^{1}|R)]+\hat{\mu }\psi \circ \eta (U,\pi_{i}^{1}|R).$$

The urban-rural inequality of opportunity in healthcare can be measured according to (9), from which we also obtain a decomposition form (the right hand of this equation), similar to what is proposed by Oaxaca (1973) [26]. The *fairness gap* can be decomposed into three parts: (1) The constant term can be regarded as a coefficient of variable I, whose value is 1 invariably. Here, we consider I as one of the elements of $e^{2}$. In this way, the former two terms can be considered as the coefficient effect of $e^{2}$, namely the “$e^{2}$ *coefficient effect*”. This means that even if the variables $e^{2}$ are identical between rural and urban, the rural-urban healthcare would still be different because the coefficients corresponding to these $e^{2}$ variables are different. So why do urban and rural residents with same $e^{2}$ characteristics still have diverse coefficients? The possible explanation is discrimination or cultural difference.

(2) The third term of this equation $\hat{\gamma }[\psi \circ \eta (U,\pi_{i}^{1}|R)-\psi \circ \eta (R,\pi_{i}^{1}|R)]$ can be regarded as the “*e*^1^ *environmental characteristic effect*”, which indicates that part of the urban-rural healthcare gap is from the difference between the counterfactual characteristics of *e*^1^—the same rural residents with the same degree of *effort* and the same *effort* distribution characteristics but in the urban *circumstances*—and its actual characteristics when holding the coefficient of e1 as constant as that of rural residents.

(3) The fourth term $\hat{\mu }\psi \circ \eta (U,\pi_{i}^{1}|R)$ can be regarded as the “*e*^1^ *environmental coefficient effect*”, which indicates that part of the urban-rural healthcare gap is from the implacable urban-rural coefficient differences of *e*^1^, even if the rural residents are endowed with the same *circumstances* as the urban and are then able to reproduce a new distribution of $e^{1}$.

The linear form is:

(10) $$hc_{i}=\alpha +\beta c_{i}+(\gamma +\mu c_{i})e_{i}^{1}+(\delta +\rho c_{i})e_{i}^{2}+\varepsilon_{i}$$

for (8), and

(11) $$e_{i}^{1}=a+bc_{i}+(d+lc_{i})\pi_{i}^{1}+\tau_{i}$$

for (6), where in (11), a is the constant; *b*, *d*, and *l* are parameters; and $\tau_{i}$ is an error item. The fairness gap is:

(12) $$f.g.=\hat{\beta }+\hat{\rho }E(e_{i}^{2}|R)+\hat{\gamma }[E(U,\pi_{i}^{1}|R)-E(R,\pi_{i}^{1}|R)]+\hat{\mu }E(U,\pi_{i}^{1}|R),$$

We use the propensity score to obtain individual *π* (degree of *effort*) in their own groups. Meanwhile, allowing the error terms of (10) and (11) to correlate, we use the Geweke-Hajivassiliou-Keane (GHK) simulation (as Gates (2007) [27] explains, the GHK simulation has excellent features, and is widely used in the health economics domain, e.g., Deb and Trivedi (2006) [28], Balia and Jones (2008) [29], Rosa-Dias (2010) [13], etc. STATA has already developed a corresponding command *cmp*, which is introduced in detail by Roodman (2009) [30]) to estimate the system [27].

## 3. Data

### 3.1. Data Sources

We use the China Health and Nutrition Survey, which is collected by the Carolina Population Center of the University of North Carolina at Chapel Hill, and the National Institute of Nutrition and Food Safety at the Chinese Center for Disease Control and Prevention (CDC). It includes 10 waves. As mentioned before, one of our major contributions is disentangling the inequality of opportunity between urban and rural residents due to separate basic medical insurance. Since the Chinese local government piloted the URIMIS from 2009, and we cannot access the county-level policy information about the URIMIS due to the confidentiality agreement of the CHNS dataset, the decomposition results would be confounded if we use the whole data waves. Therefore, this paper only uses data waves from 1997 to 2006, and the healthcare EOp situations after 2009 are analyzed in our other papers by using other suitable datasets. We set wave 1997 and 2000 as the *period 1* group and waves 2004 and 2006 as the group of *period 2* because the NCMS was established in 2003.

### 3.2. Variables

We employ the healthcare expenditure of the past four weeks as healthcare utilization. By reference of relative studies on racial/ethical disparities of health and healthcare [19,28,29,30], we define that the $e^{1}$-vector of illegitimate factors includes medical insurance policy indicator, region and socioeconomic status (SES), etc., and that the $e^{2}$-vector of legitimate factors includes healthcare needs and individual preferences. Variables in $e^{1}$ can be classified into three parts: (1) SES variables, including family per capita income (yuan/year) and formal education (year), (2) policy variable, i.e., reimbursement ratio (%), (3) healthcare environmental variables, including region (1 = the east region, 0 = others), medicine availability (Is there a health facility nearby? 1 = yes, 0 = no) and travel time by bike to the health facility (min). Variables in $e^{2}$ are classified into four parts: (1) demographic variables, including age, sex (1 = male, 0 = female), and marital status (1 = married, 0 = others), (2) general health variables, including self-reported health status (4 = excellent, 3 = good, 2 = fair, 1 = poor) and chronic disease history (1 = yes, 0 = no), (3) health variables reflecting situations of illness, i.e., types of illness one had suffered from and the severity of the illness, during the past four weeks (3 = quite severe, 2 = somewhat severe, 1 = not severe), (4) preference variables, including treatment preferences (What to do when ill? 4 = none, 3 = saw a doctor, 2 = saw the local health worker, 1 = self-care) and lifestyle preferences, such as whether they smoke or drink (1 = yes, 0 = no).

The actual reimbursement ratio (the proportion of healthcare expenditure paid for by medical insurance (in the CHNS questionnaires, there are relevant questions which we can use directly for the measurement)) is the best indicator to measure the insuring degree and the healthcare economic burden. If respondents have no medical insurance and have spent zero on healthcare, we take their self-reported policy reimbursement ratios (in the CHNS questionnaires, the corresponding questions are: “What percentage of the fees for outpatient care does your insurance pay (not including registration fee)”and “What percentage of the fees for inpatient care does your insurance pay (not including food expenses)”) as value. If one’s self-reported policy reimbursement ratio is missing, we replace the missing value with the average value of respondents with the same characteristics. Meanwhile, since the treatment preferences are important in healthcare research, we control for treatment preferences to some extent via the answer to “what did you do when you felt ill”. In addition, price level adjusts to the year 2009.

The final sample includes 4168 observations. *Period 1* includes 1076 individuals, and *period 2* includes 3092. In *period 1*, 412 respondents are from urban areas. There are 1283 urban respondents and 1809 rural respondents in *period 2*, respectively. The description of variables is shown in Table 1, where we can see obvious urban-rural differences in the past four-week healthcare expenditure. The value of differences are 225.096 and 268.149, respectively, in *period 1* and *period 2*, and the urban residents are found to be higher in both periods. Urban-rural differences in SES variables, income, and education are huge too, as well as policy variable actual reimbursement ratio.

## 4. Results

How large are the urban-rural inequalities of opportunity in health care utilization? The results are shown in Table 2.

Setting urban *circumstances* as the “baseline” reference *circumstance* of c, the total *fairness gap* is 262.670 yuan in *period 1*, and the urban-rural difference is 225.096 yuan. The ratio of the *fairness gap* is 1.167 in *period 1*. The statistics shows that urban residents spend 100 yuan more than rural residents. However, rural residents should spend 16.7 yuan per capita more than urban residents under the equity view. The *fairness gap* will reach 116.7 yuan. Similarly, the urban-rural difference is 268.149 yuan in *period 2*. The calculated *fairness gap* is 467.521 yuan. The ratio of the fairness gap to the average urban-rural difference is 1.744. The statistics shows that rural residents spend 100 yuan per capita less than urban residents. Based on the EOp, rural residents should spend 74.4 yuan per capita more than urban residents. Compared with the outcome inequality, the situation of rural residents is much worse. Moreover, the value of the *fairness gap* in *period 2* is bigger than *period 1*. It shows that the inequality of opportunity increases with time.

Table 2 also shows the three parts of the *fairness gap*. The effect of the first part is more significant than that of the other two. The ratios of *e*^2^ *coefficient effects* are 0.674 in *period 1* and 1.173 in *period 2*. This indicates that the urban-rural gaps accounts for 57.75% of the whole *fairness gap* in *period 1*, and 67.25% in *period 2*. We think that health consciousness and service qualities are an ingrained difference between urban and rural residents. Urban residents have more economic resources and prefer to invest more in health. Advanced medical technology is also first applied in urban areas. The *e1 environmental coefficient effect* is 8.5% in *period 1*, and 4.8% in *period 2*. In addition, the ratios of *e*^1^ *environmental characteristic effect* are 0.408 and 0.522, respectively. It makes up 34.96% of the whole *fairness gap* in *period 1* and 29.93% in *period 2*.

There is an index number problem in the Oaxaca decomposition. Based on De Murger et al. (2007) [31], we re-conduct the *fairness gap* decomposition, with rural *circumstances* (R) as the “baseline” reference *circumstances*. The robustness test supports the above results. The details are shown in Table 3.

## 5. Discussion

This study aimed to measure the urban-rural inequality of opportunity in healthcare in China based on the theory of Equality of Opportunity. In order to assess whether there was an increase or decrease in this inequality gap, we examined the reimbursement ratio within the medical insurance system with a particular focus on the fairness gap between *period 1* (1997–2000) and *period 2* (2004–2006). Empirical analysis using CHNS data shows that the ratios of the *fairness gap* to the directly observed average urban-rural difference in healthcare are 1.167 during *period 1* and 1.744 during *period 2*. The average urban-rural difference observed directly from original statistical data may underestimate the degree of essential inequity.

Moreover, we also find that the reimbursement ratio predicts a dramatic change of effect between *period 1* and *period 2*. In this discussion section, we take a look at the *fairness gap* generating process caused by the reimbursement ratio, and Table 2 shows an interesting change. The reimbursement ratio, which plays a big role in *period 1* (the ratio to the average difference is 0.236), shows little importance (the ratio is as little as 0.003) in *period 2*. One possible interpretation is that *period 1* is before the establishment of the NCMS, when many rural residents lacked sufficient and efficient medical insurance, and when participating in only certain medical insurances (e.g., the UEBMI). Straightly speaking, enjoying a certain reimbursement for outpatient or inpatient services represented a kind of privilege, especially for urban residents. This is very important because the privilege in healthcare usually relates to better health services and higher prices. On the one hand, privilege encourages insureds to seek health care. On the other hand, those that are not insured, especially poor rural residents, are reluctant to purchase healthcare, unless seriously ill. As urban residents—especially urban workers and government officers—and a few rich rural residents get most of the privilege, the urban-rural *fairness gap* expressed by the reimbursement ratio cannot be overlooked. However, during *period 2*, the NCMS was already established, and more rural residents participated and enjoyed the benefits of the reimbursement. Reimbursement ratio is no longer a privilege for urban residents. Therefore, the effect of the reimbursement ratio becomes so small that we can ignore it for *period 2*.

We also observe an increasing fairness gap between *period 1* and *period 2* in Table 2, and this increasing trend is faster than that of the urban-rural difference. Although there is a clear rise in reimbursement ratio for rural residents (shown in Table 1), the effect reimbursement ratio has on narrowing of the *fairness gap* is rather small.

As mentioned above, many rural residents, especially the rural poor, lacked sufficient and efficient medical insurance and healthcare in *period 1* since the NCMS was not established yet. As a result, healthcare consumption in rural areas is a kind of passive consumption. For most rural residents, going to the hospital is the last resort. Therefore, income is almost irrelevant to healthcare expenditure in this period. However, in *period 2*, as the NCMS had already been established, more and more rural residents had participated in medical insurance. The health-seeking behaviors and health perceptions changed gradually among rural residents, and healthcare consumption became more and more positive. Therefore, the influence of income on the *fairness gap* increased (from −0.069 to 0.548; see Table 2), and income became an important factor for the *fairness gap* change. Since in recent years, the urban-rural income gap has been widening (see Table 1), we believe that the trend of the increasing urban-rural income gap should be taken into consideration when improving medical insurance policies. Although the medical insurance system does not seek to narrow the urban-rural income gap, such income gap has already worsened the performance of medical insurance policies.

There is no doubt that the relevant government sectors, which are responsible for the medical insurance policy making and supervision, have made great effort to narrow the urban-rural reimbursement difference. However, since the urban-rural income gap is widening, such effort may be counterproductive. Just as described in Example C in Appendix A, the income gap can only counteract the good intentions of current medical insurance policies, being a hindrance for URIMIS aims. Therefore, only generally leveling reimbursement ratios between urban and rural residents is now obviously insufficient to mitigate the urban-rural inequalities in healthcare, under the background of the income-gap widening. On the basis of Roemer’s EOp, pro-disadvantage policies on reimbursement are highly desiderated.

Rural residents have made great contribution to China’s economic development. However, what they share from the prosperity is far less than what they should obtain. The inequality in healthcare is just one conspicuous aspect among the urban-rural illegitimate gaps. Since the 21st century, China has been improving rural health and healthcare conditions with great efforts, including the expansion of the NCMS and increase in NCMS reimbursement ratios. Surprisingly, most of the previous literature found that the NCMS was not able to significantly reduce the medical burden for rural residents in China [1,2,31]. The exploration of the URIMIS is being heatedly discussed, but without an agreement on how to effectively reduce or even eliminate the urban-rural disparities in healthcare. This paper suggests that focusing on the urban-rural inequalities of opportunity is much more meaningful than focusing on the urban-rural outcome equalities or reimbursement equalities in health care. Generally leveling the reimbursement ratios between urban and rural residents is not sufficient to realize the EOp in healthcare; pro-disadvantage reimbursement policies are needed.

There inevitably are some limitations in our research. The decomposition strategy may need further modification for accuracy. Nevertheless, as mentioned before, this paper is a preliminary study, using the theory of the EOp, on China’s special medical insurance reimbursement policies, in order to provide some useful suggestions on the further improvement of medical insurance systems. In addition, although we want to examine the time trend of the inequality of opportunity in healthcare in China after 2009, we have to give up due to data limitations. The CHNS stopped providing a self-assessed health level after 2009 and we cannot access county-level policy information about the URIMIS due to a confidentiality agreement in the CHNS dataset; the decomposition results would be confounded if we used the entire waves of the data. We hope that this paper will inspire more interest in the conceptualization and measurement of urban-rural healthcare justice in China.

## 6. Conclusions

Based on EOp and the *compensation principle*, this paper analyzes inequality in healthcare utilization between urban and rural China. We define three components of the *fairness gap*, the *e*^2^ *coefficient effect, the e1 environmental characteristic effect,* and the *e*^1^ *environmental coefficient effect*. The results indicate that statistical data may underestimate the degree of essential inequalities, which are from environmental characteristics. Inequality of opportunity is mainly from the expansion of income inequality, especially “hukou” restrictions. With NCMS implementation, the effect of reimbursement ratio had a dramatic change during the two periods as it reduces the inequalities of opportunity between urban and rural settings.

Due to widening of the urban-rural income gap, it is insufficient to narrow the fairness-gap only by unifying the medical insurance policies for both urban and rural residents. In accordance with the maximin principle of Roemer, our suggestion is that we improve the affordability of the rural poor. For example, first, the government should promote rural-urban migration and local rural urbanization, which will make medical care easily accessible to rural households. Second, the central government needs to settle medical expenses, where they are incurred via basic medical insurance accounts, especially for rural households. Third, the government also needs to increase medical aid for major illnesses in rural households. Moreover, it also can increase pension to alleviate the burden of the rural poor through the income effect. Overall, this study highlights the widening gap in inequality of opportunity over two time periods, and the urgent need to strengthen healthcare services in rural China in a bid to narrow the gap.

## Figures and Tables

**Table 1:** Description of Variables.

Variables	*Period 1*				*Period 2*			
	Urban		Rural		Urban		Rural	
	Mean	Sd.	Mean	Sd.	Mean	Sd.	Mean	Sd.
*Y*								
Healthcare expenditure over the past four weeks	779.758	2201.553	554.663	2189.791	709.827	5039.766	441.677	2351.327
*e*^1^								
Family per capita income (yuan/year)	6943.783	7030.383	4569.000	5328.661	10,729.150	10,548.700	5796.847	8870.297
Formal education years	7.124	4.730	5.066	4.031	7.836	4.819	5.516	4.181
Reimbursement ratio (%)	26.036	37.793	6.143	23.223	25.116	34.834	9.360	24.019
Region (1 = the east region, 0 = others)	0.383	0.487	0.325	0.469	0.486	0.500	0.411	0.492
Travel time (min) by bike to health facility	17.197	20.373	16.089	18.706	14.499	14.464	13.439	17.789
Medicine availability (1 = yes, 0 = no)	0.951	0.215	0.967	0.179	0.988	0.111	0.985	0.121
*e*^2^								
*Basic Demographic Information*								
Age (years)	53.008	16.252	52.322	15.692	54.145	15.897	55.435	14.686
Sex (1 = male, 0 = female)	0.422	0.495	0.438	0.497	0.434	0.496	0.423	0.494
Marital status (1 = married, 0 = others)	0.801	0.400	0.797	0.403	0.796	0.403	0.811	0.392
*General Health Information*								
Self-reported health status (4 = excellent, 3 = good, 2 = fair, 1 = poor)	2.138	0.750	2.056	0.819	2.228	0.797	2.061	0.785
Ever diagnosed with high blood pressure (1 = yes, 0 = no)	0.182	0.386	0.148	0.355	0.246	0.431	0.170	0.376
Diabetes (1 = yes, 0 = no)	0.158	0.365	0.123	0.329	0.194	0.396	0.132	0.339
Myocardial infarction (1 = yes, 0 = no)	0.015	0.120	0.014	0.116	0.014	0.118	0.009	0.094
Apoplexy (1 = yes, 0 = no)	0.039	0.193	0.027	0.163	0.034	0.180	0.025	0.156
*Illness During the Past Four Weeks*								
Suffered from chronic or acute diseases (1 = yes, 0 = no)	0.874	0.332	0.883	0.322	0.634	0.482	0.669	0.471
Had fever, sore throat, or cough (1 = yes, 0 = no)	0.359	0.480	0.357	0.479	0.373	0.484	0.362	0.481
Had diarrhea or stomach ache (1 = yes, 0 = no)	0.126	0.332	0.131	0.338	0.156	0.363	0.153	0.360
Had headache or dizziness (1 = yes, 0 = no)	0.306	0.461	0.283	0.451	0.253	0.435	0.265	0.441
Had joint pain or muscle pain (1 = yes, 0 = no)	0.165	0.372	0.181	0.385	0.260	0.439	0.281	0.450
Had rash or dermatitis (1 = yes, 0 = no)	0.032	0.175	0.024	0.153	0.036	0.186	0.024	0.152
Had eye/ear disease (1 = yes, 0 = no)	0.034	0.181	0.026	0.158	0.062	0.240	0.050	0.217
Had heart disease/chest pain (1 = yes, 0 = no)	0.102	0.303	0.069	0.254	0.112	0.316	0.082	0.274
Had other infectious disease (1 = yes, 0 = no)	0.032	0.175	0.032	0.175	0.047	0.211	0.050	0.217
Had a non-communicable disease (1 = yes, 0 = no)	0.158	0.365	0.149	0.356	0.244	0.430	0.187	0.390
Severity of the illness (3 = quite severe, 2 = somewhat severe, 1 = not severe)	1.740	0.689	1.640	0.674	1.687	0.657	1.702	0.665
Inpatient visits (1 = yes, 0 = no)	0.092	0.290	0.074	0.262	0.031	0.174	0.030	0.170
*Preferences*								
What to do when ill (4 = none, 3 = saw a doctor, 2 = saw the local health worker, 1 = self-care)	2.522	0.908	2.706	0.751	2.074	1.168	2.472	1.048
Ever smoked (1 = yes, 0 = no)	0.250	0.434	0.304	0.460	0.313	0.464	0.307	0.462
Drink alcohol last year (1 = yes, 0 = no)	0.316	0.465	0.280	0.449	0.341	0.474	0.280	0.449
**Number of sub-samples**	412		664		1283		1809	

Note: “Sd.” denotes standard deviation.

**Table 2:** Decomposition of Fairness Gaps Using CHNS Data.

	*Period 1*		*Period 2*	
Directly observed average differences	225.096		268.149	
	Fairness Gap	Ratio	Fairness Gap	Ratio
***e*^2^*coefficient effect*:**				
Age	387.248	1.720	−801.470	−2.989
Male	76.158	0.338	251.163	0.937
Married	−123.302	−0.548	−105.519	−0.394
Self-reported health: fair	166.831	0.741	−593.614	−2.214
Self-reported health: good	56.920	0.253	−305.803	−1.140
Self-reported health: excellent	18.001	0.080	−29.494	−0.110
High blood pressure	54.396	0.242	0.897	0.003
Diabetes	−79.090	−0.351	51.664	0.193
Myocardial infarction	6.067	0.027	−4.507	−0.017
Apoplexy	7.781	0.035	−3.898	−0.015
Suffered from chronic or acute diseases	667.391	2.965	−165.665	−0.618
Had fever, sore throat or cough	−136.195	−0.605	90.036	0.336
Had diarrhea or stomachache	−28.023	−0.124	−44.276	−0.165
Had headache or dizziness	78.907	0.351	−48.257	−0.180
Had joint pain or muscle pain	−1.126	−0.005	41.766	0.156
Had rash or dermatitis	4.013	0.018	40.411	0.151
Had eye/ear disease	−42.756	−0.190	24.671	0.092
Had heart disease/chest pain	34.711	0.154	63.017	0.235
Had other infectious disease	39.230	0.174	17.924	0.067
Had a non-communicable disease	−64.060	−0.285	46.340	0.173
Severity of the illness: somewhat severe	111.614	0.496	−4.149	−0.015
Severity of the illness: quite severe	82.171	0.365	133.673	0.499
Inpatient	−8.388	−0.037	43.000	0.160
See local health worker when ill	0.692	0.003	−20.647	−0.077
See a doctor when ill	163.488	0.726	189.518	0.707
Do nothing when ill	31.194	0.139	55.693	0.208
Smoke	−3.613	−0.016	−54.234	−0.202
Drink	−165.846	−0.737	−103.918	−0.388
Wave	−27.906	−0.124	−99.114	−0.370
Intercept	−1154.844	−5.130	1649.307	6.151
	**151.663**	**0.674**	**314.513**	**1.173**
***e*^1^ *environmental characteristic effect:***				
Family per capita income	−15.542	−0.069	147.014	0.548
Education	70.475	0.313	13.324	0.050
Reimbursement ratio	53.126	0.236	0.793	0.003
East China	−8.402	−0.037	−20.763	−0.077
Travel time to health facility	−9.245	−0.041	−0.729	−0.003
Medicines available	1.352	0.006	0.373	0.001
	**91.763**	**0.408**	**140.011**	**0.522**
***e*^1^*environmental coefficient effect:***				
Family per capita income	−28.221	−0.125	87.714	0.327
Education	129.135	0.574	16.578	0.062
Reimbursement ratio	16.633	0.074	10.050	0.037
East China	−34.839	−0.155	−64.626	−0.241
Travel time to health facility	−68.028	−0.302	21.779	0.081
Medicines available	4.564	0.020	−58.499	−0.218
	**19.244**	**0.085**	**12.997**	**0.048**
**Total**	**262.670**	**1.167**	**467.521**	**1.744**
**Number of sub-samples**	1076		3092	

Note: “Ratio” in the third and fifth columns denotes the ratio of the decomposed *fairness gap* as well as the total *fairness gap*, i.e., each cell in the second and fourth column, to the directly observed average difference in the corresponding period.

**Table 3:** Robustness Test of Table 2; Rural as the Reference Circumstance.

	*Period 1*		*Period 2*	
Directly observed average differences	225.096		268.149	
	Fairness Gap	Ratio	Fairness Gap	Ratio
***e*^2^*coefficient effect*:**				
Age	392.324	1.743	−782.817	−2.919
Male	73.391	0.326	257.510	0.960
Married	−123.965	−0.551	−103.547	−0.386
Self-reported health: fair	192.867	0.857	−556.721	−2.076
Self-reported health: good	61.505	0.273	−410.789	−1.532
Self-reported health: excellent	13.187	0.059	−39.637	−0.148
High blood pressure	67.092	0.298	1.297	0.005
Diabetes	−101.040	−0.449	75.892	0.283
Myocardial infarction	6.518	0.029	−7.149	−0.027
Apoplexy	11.146	0.050	−5.252	−0.020
Suffered from chronic or acute diseases	660.779	2.936	−156.815	−0.585
Had fever, sore throat or cough	−137.071	−0.609	92.643	0.345
Had diarrhea or stomachache	−26.994	−0.120	−45.075	−0.168
Had headache or dizziness	85.231	0.379	−46.166	−0.172
Had joint pain or muscle pain	−1.028	−0.005	38.642	0.144
Had rash or dermatitis	5.254	0.023	60.954	0.227
Had eye/ear disease	−56.747	−0.252	30.534	0.114
Had heart disease/chest pain	51.078	0.227	86.451	0.322
Had other infectious disease	39.139	0.174	16.848	0.063
Had a non-communicable disease	−67.785	−0.301	60.327	0.225
Severity of the illness: somewhat severe	122.736	0.545	−4.157	−0.016
Severity of the illness: quite severe	103.797	0.461	124.753	0.465
Inpatient	−10.483	−0.047	44.911	0.167
See local health worker when ill	0.367	0.002	−8.647	−0.032
See a doctor when ill	151.617	0.674	124.701	0.465
Do nothing when ill	26.976	0.120	53.760	0.200
Smoke	−2.969	−0.013	−55.150	−0.206
Drink	−186.812	−0.830	−126.831	−0.473
Wave	−24.483	−0.109	−97.180	−0.362
Intercept	−1154.844	−5.130	1649.307	6.151
	**170.783**	**0.759**	**272.597**	**1.017**
***e*^1^*environmental characteristic effect:***				
Family per capita income	−0.874	−0.004	72.381	0.270
Education	18.030	0.080	6.352	0.024
Reimbursement ratio	16.448	0.073	−16.123	−0.060
East China	−2.170	−0.010	−9.087	−0.034
Travel time to health facility	−4.561	−0.020	−2.447	−0.009
Medicines available	2.793	0.012	0.519	0.002
	**29.667**	**0.132**	**51.594**	**0.192**
***e*^1^*environmental coefficient effect:***				
Family per capita income	−42.889	−0.191	162.347	0.605
Education	181.580	0.807	23.550	0.088
Reimbursement ratio	53.310	0.237	26.966	0.101
East China	−41.072	−0.182	−76.301	−0.285
Travel time to health facility	2.187	0.010	−58.644	−0.219
Medicines available	−72.712	−0.323	23.497	0.088
	**80.404**	**0.357**	**101.414**	**0.378**
**Total**	**280.854**	**1.248**	**425.605**	**1.587**
**Number of sub-samples**	1076		3092	

Note: “Ratio” in the third and fifth columns denote the ratio of the decomposed *fairness gap* as well as the total *fairness gap*, i.e., each cell in the second and fourth columns, to the directly observed average difference in the corresponding period.

## Data Availability

This research uses data from China Health and Nutrition Survey (CHNS). It is available from: https://www.cpc.unc.edu/projects/china.

## Appendix A

Examples of the misleading aspects of outcome equality in healthcare analysis.

In the introduction, we endorsed the idea of focusing on essential equity, i.e., the equality of opportunity (EOp), rather than the outcome equality or reimbursement equality of healthcare. Here, we give three examples as a simple explanation. Example A and B explain the misleading use of the outcome equality, and Example C, the reimbursement equality.

Example A: Suppose the aging proportion is higher among urban residents, who involuntarily have more health needs, and thus more health care expenditure than the rural residents. Such urban-rural differences due to demographic characteristics are indeed reasonable and desirable, reflecting the effective allocation of health resources. Under such situation, policies need not interfere, while purchasing outcome equality may result in inefficiency.

Example B: Suppose there are two residents belonging to urban and rural areas, respectively. The healthcare expenditure of the rural resident should have been 1000 yuan because of a serious illness. However, owing to lack of money or effective medical security, their actual expenditure is only 500 yuan. Meanwhile, the urban resident, who enjoys a more generous medical insurance, spends the same 500 yuan for a health problem, such as flu, which could have been cured at the expense of only 100 yuan. There is no straightforward inequality visible here from the aspect of actual expenditure on healthcare. However, essential inequality is concealed.

Example C: Suppose there are two residents belonging to urban and rural areas, respectively, and enjoying the same reimbursement of 50%. One day, both of them are stricken by the same disease, such as flu. However, the rural resident decides not to see a doctor because of lack of money, but the urban resident does. Then, the premium paid by the rural resident in effect is used to reimburse the urban resident, resulting in the phenomenon of the rural helping the urban or the poor helping the rich, although we are reluctant to face the truth. Thus, when we judge based on reimbursement equality, such as unifying the reimbursement policies for both urban and rural residents, there may also be essential inequalities.
